# The impact of *Schistosoma haematobium* hybridization on molecular diagnosis of schistosomiasis: A review with emphasis on female genital schistosomiasis

**DOI:** 10.1371/journal.pntd.0013364

**Published:** 2025-08-07

**Authors:** Ombeni Ally, Bernard N. Kanoi, Gideon S. Mmbando, Steven Ger Nyanjom, Ladslaus L. Mnyone, Jesse Gitaka, Gerald Misinzo, Lucy Ochola

**Affiliations:** 1 Department of Biology, College of Natural and Mathematical Sciences, University of Dodoma, Dodoma, Tanzania; 2 Directorate of Research and Innovation, Mount Kenya University, Thika, Kenya; 3 Department of Biochemistry, Jomo Kenyatta University of Agriculture and Technology, Nairobi, Kenya; 4 Institute of Pest Management, Sokoine University of Agriculture, Morogoro, Tanzania; 5 SACIDS Africa Centre of Excellence for Infectious Diseases, Sokoine University of Agriculture, Morogoro, Tanzania; 6 Department of Tropical and Infectious Diseases, Institute of Primate Research, Nairobi, Kenya; George Washington University School of Medicine and Health Sciences, UNITED STATES OF AMERICA

## Abstract

Female genital schistosomiasis (FGS) is a gynecological manifestation of urinary schistosomiasis in female genitals. FGS is a neglected tropical disease; not only are most patients unaware of the condition, but healthcare workers and policymakers have inadequate knowledge about it. The treatment and control of FGS relies on current guidelines for controlling and eliminating schistosomiasis without rigorous focus on clinical evidence of the presence of FGS. Neglect of FGS has led to the misconception that the disease is sexually transmitted. Diagnosing FGS remains challenging as there is no widely accepted reference assay. Urine examination, which is the gold standard in urogenital schistosomiasis has some limitations in diagnosing FGS as the demonstration of *Schistosoma haematobium* and/or eggs alone does not necessarily indicate FGS. In order to overcome challenges with the biopsy and colposcopy approach, some studies have evaluated the potential of PCR-based assays and isothermal amplification of *Schistosoma* DNA. Recent studies have reported hybridization between *S. haematobium* and other livestock schistosomes, but little is known about the impact of hybridization on schistosomiasis diagnosis. These hybrids not only affect livestock and humans but also have their genomes modified, and in some cases, abnormal egg morphology due to *Schistosoma* hybridization might affect the actual prevalence estimation. Herein, we highlight the potential impacts of *S. haematobium* hybridization on molecular diagnosis of schistosomiasis, with an emphasis on FGS.

## Methods

**Fig 1:**
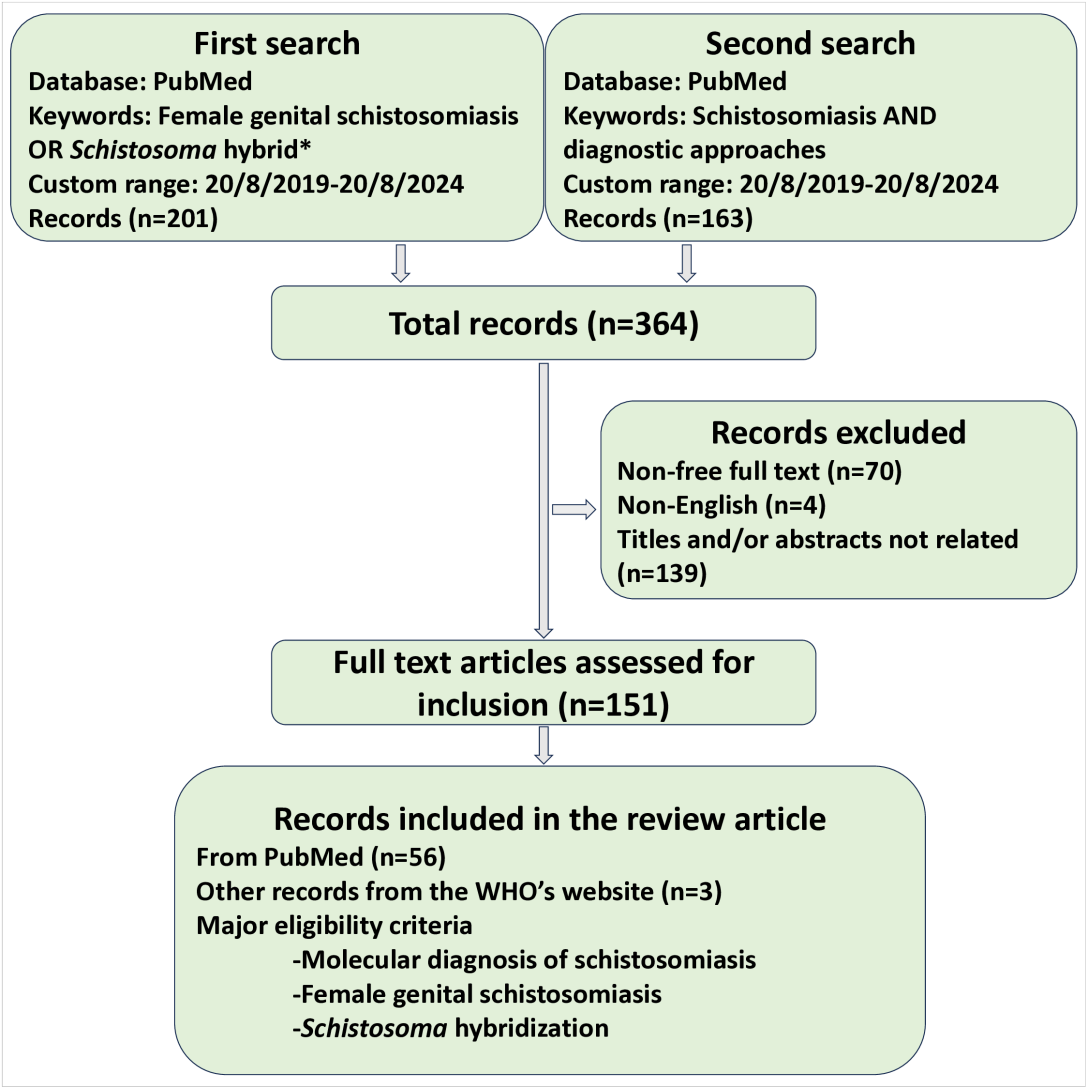
Flowchart of the literature search and selection of studies.

The aim of this review was to examine the impact of *Schistosoma* hybridization on the molecular diagnosis of schistosomiasis, with emphasis on female genital schistosomiasis (FGS). We used the PubMed search engine to identify peer-reviewed articles related to FGS and *Schistosoma* hybridization (Fig 1). The Boolean search terms “female genital schistosomiasis OR *Schistosoma* hybrid*” were applied to retrieve articles published in English between August 20, 2019, and August 20, 2024. We selected both research and review articles focusing on *Schistosoma* hybridization and/or FGS for qualitative narrative analysis. To supplement the discussion on FGS diagnostics, an additional PubMed search was conducted using the Boolean terms “schistosomiasis AND diagnostic approaches”, restricted to the same publication period as the primary search. A total of 364 records were retrieved from both searches. Of these, 56 articles that addressed at least one of the core topics: FGS, *S. haematobium* hybridization, or molecular diagnosis of schistosomiasis were included in the final review. In addition, three relevant records were sourced from the World Health Organization (WHO) website.

## Introduction

Schistosomiasis, also known as bilharzia, is one of the neglected tropical diseases that affect both humans and animals. Tropical and sub-tropical areas are home to several *Schistosoma* species that affect both wild and domestic animals. *Schistosoma magrebowiei*, *S. rodhaini*, *S. spindale*, *S. indica*, *S. incognitum*, *S. nasale*, and *S. leiperi* mostly affect wild animals, while *S. bovis*, *S. curasoni*, and *S. mattheei* are associated with schistosomiasis in domestic animals [1,2]. The disease in humans is caused by six major schistosomes: *S. mansoni*, *S. japonicum*, *S. mekongi*, *S. guineensis*, *S. intercalatum*, and *S. haematobium* [2,3]. The disease affects at least 240 million people worldwide, manifesting either as intestinal or urogenital, predetermined by the causative species [2,4]. Schistosomiasis primarily affects low-income rural communities with poor access to clean water, sanitation, and healthcare, and can cause disability-adjusted life years [2]. Sub-Saharan Africa, home to about 13% of the global population, bears nearly 90% of schistosomiasis cases, resulting in an estimated 280,000 annual deaths from the disease [5].

*S. haematobium*, which causes urogenital schistosomiasis, is one of the two main schistosome species that are prevalent in Africa [2]. At low intensities, *S. haematobium* infection causes treatable and reversible nutritional impairment and tissue reactions, leading to hematuria, frequent and painful urination, and urinary tract infections [6,7]. In cases of heavy infection, the disease results in permanent anatomical changes, such as fibrosis, bladder cancer, and damage to the genital tract [6].

In females, *S. haematobium* eggs affect the reproductive system, causing a condition known as FGS. FGS is a gynecological manifestation of urinary schistosomiasis in female genitals [8]. Estimates show that FGS affects at least 56 million women and girls worldwide; almost all of them originate from Africa [2,4]. Surprisingly, FGS is one of the most neglected tropical diseases; not only are patients unaware of the condition, but healthcare workers and policy makers have inadequate knowledge about it.

FGS-associated complications have impacts towards the elimination of other diseases. Several studies have reported positive synergies between FGS and the acquisition of viruses, such as the human immunodeficiency virus (HIV) [9–11] and the human papillomavirus (HPV) [11,12]. In HIV acquisition, the intracervical lesions caused by FGS act as a door for HIV when an affected female is having sex with an HIV-infected man. This, in turn, increases the likelihood of HIV transmission in communities with a high prevalence of FGS.

Currently, the diagnosis of FGS relies on combination of clinical manifestation of FGS and visual examination of *Schistosoma* eggs and/or *Schistosoma* eggs-induced lesions [4,13]. Visual examination using biopsy and colposcopy approach offers reliable specificity, but these assays are not widely accepted. Subsequently, researchers are exploring the use of DNA amplification, specifically isothermal assays to replace the existing diagnostic assays for FGS. However, the recent reports regarding the hybridization of *S. haematobium* with the livestock schistosomes might complicate these initiatives.

Several studies in Southern Europe, Nigeria, Ivory Coast, Niger, Benin, Mali, and Senegal have reported the genetic hybridization of *S. haematobium* with closely related livestock schistosomes, such as *S. bovis* and *S. currasoni* [14–18]. These hybrids not only affect livestock and humans, but having their genomes modified, and in some cases, abnormal egg morphology due to *Schistosoma* hybridization might affect the actual prevalence estimation [14,15]. This review highlights the challenges in diagnosing FGS and the potential impact of *S. haematobium* hybridization.

## Female genital schistosomiasis

Human urogenital schistosomiasis can manifest as both acute and chronic infections [19]. In either case, schistosomes can induce a complex immune reaction due to the presence of adult schistosomes or their eggs. Immunological reactions to deposited schistosome eggs in female reproductive organs result in painful and stigmatizing symptoms such as vaginal discharge, itching, leucorrhea, contact bleeding, abdominal pain, menstrual cycle abnormalities, dyspareunia, and in some cases, unbearable pain during sexual intercourse are the most common symptoms which collectively represent FGS [19,20]. FGS mostly affects people in sub-Saharan Africa, and affects at least 56 million young ladies and women [4]. In pregnant women, FGS is associated with stillbirth, ectopic pregnancy, and miscarriage, but the actual pathological mechanism remains unclear [10,19]. In highly prevalent areas, FGS-associated complications have impacts towards the elimination of other diseases. Several studies have reported positive synergies between FGS and the acquisition of viruses, HIV/AIDS, and HPV [9,10,12]. A study conducted in Madagascar reported a prevalence of 62.6% (*n* = 189; 95% CI: 56.9–68.1) and 42.7% (*n* = 129; 95% CI: 37.1–48.5) for FGS and HPV, respectively [12]. In HIV acquisition, the intracervical lesions caused by FGS act as a door for HIV when a female is having sex with an infected man [10]. This, in turn, increases the likelihood of HIV transmission in communities with a high prevalence of FGS.

## Transmission

**Fig 2:**
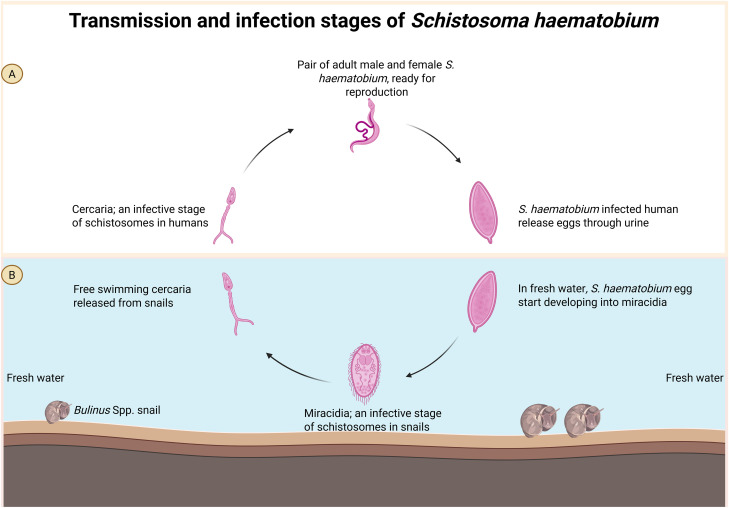
Transmission and infection stages of *Schistosoma haematobium.* **A**: in humans, and **B**: in snails. Created in BioRender.Com.

The FGS’s negligence in healthcare sector and education system has led to the misconception that the disease is sexually transmitted [21]. Most people, including healthcare practitioners and infected individuals, either lack knowledge about the etiology and transmission of FGS or mistakenly believe that unprotected sexual intercourse transmits the disease from one infected person to another [21]. However, this myth has been debunked as FGS is a freshwater-born disease [8], which is transmitted by the *Bulinus* spp. snails (Fig 2).

Women and young ladies acquire FGS infections through contact with *S. haematobium*-contaminated freshwater bodies such as rivers, lakes, streams, and dams [8]. Intermediate freshwater snails release free-swimming *S. haematobium* cercaria into contaminated water bodies. When humans contact the contaminated water, cercaria penetrate the skin, enter the human body, lose tail, and transform to schistosomula, and subsequently migrate to different parts and organs through the blood circulation. Women and young ladies in rural areas, who are particularly involved in agriculture, recreational swimming, cleaning, washing, and fetching water for domestic use, are at a high risk of contracting the disease.

## The interconnection between the life cycle of *S. haematobium* and pathogenesis of FGS

**Fig 3:**
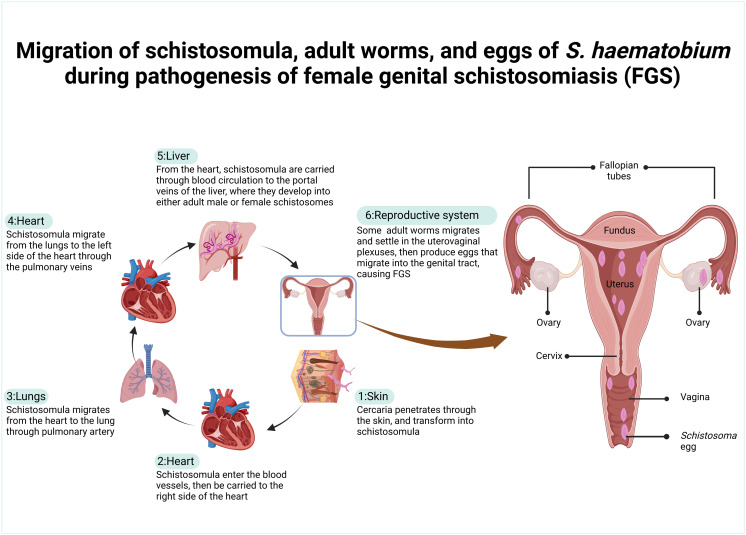
Migration pathways of *Schistosoma haematobium* through human organs. Created in BioRender.Com.

The life cycle of *S. haematobium* involves an intermediate snail and a definitive human host. In human hosts, the cycle began when free-swimming cercaria penetrated the human skin, then transformed to schistosomula. Approximately a week after cercaria penetration, the schistosomula enter the blood vessel, travel through the right side of the heart, and then enter the lungs (Fig 3) [8].

Thereafter, they migrate from the lung back to the heart through pulmonary veins, then enter the abdominal aorta. Schistosomula can pass through the celiac trunk or iliac arteries to enter the liver’s portal veins from the abdominal aorta [8]. In the liver, schistosomula loses its migratory ability and begins to grow and develop into either adult male or female *S. haematobium*. Adult male and female *S. haematobium* pair up and then move against the flow of blood in the veins, usually down the splenic and mesenteric veins. Once they get there, the paired schistosomes enter the anorectal venous plexus and then move on to the vesical venous plexus and uterovaginal venous plexus. The adult schistosomes that settle in the uterovaginal plexus produce eggs that enter the female genitals, including the cervix, vagina, uterus, and fallopian tube [19]. These eggs are capable of penetrating the walls of blood vessels in female genitals to enter the genital tract and bladder.

Eggs that are trapped in the genital tracts stimulate the immune response, causing an influx of immune cells, including neutrophils, macrophages, eosinophils, lymphocytes, plasma cells, Langerhans, histiocytes, and fibroblasts [19]. Subsequently, the inflammatory response results in granulomatous formation surrounding the schistosome eggs, thereby causing pathological changes associated with FGS [19]. The abnormal mucosal changes in FGS are responsible for the clinical manifestations of FGS, causing the development of lesions and abnormal blood vessels on the vagina, cervix, and, in some cases, vulva [4]. FGS lesions cause clinical features like irritation, itching, and abnormal vaginal discharge; bleeding and spotting; and pain during sex.

Eggs that enter the bladder are passed out through urine, thereby contributing to the continued transmission of schistosomiasis. When these eggs get into contact with freshwater, they hatch into miracidia. The free-swimming miracidia infect the *Bulinus* spp. snail [22,23], then undergo asexual reproduction to form free-swimming cercaria, which is the infective stage of schistosomes in definitive human hosts.

## Challenges in FGS diagnosis

The diagnosis of FGS remains challenging as there is no widely accepted reference assay [19]. Urine examination, which is the gold standard in urogenital schistosomiasis and *Schistosoma* antigen-based assays have some limitations in diagnosing FGS as the demonstration of *S. haematobium* and/or eggs alone does not necessarily indicate FGS (Table 1).

**Table 1:** Diagnostic approaches for demonstrating the presence of circulating schistosomes and/or eggs in humans and whether the assay can be used for FGS or not.

No	Diagnostic approach	Advantages	Disadvantages	References
01	Microscopy	• Gold standard for urogenital schistosomiasis • Much more specific in detecting schistosomes	• Demonstrating *Schistosoma* eggs does not necessarily indicate FGS • Examination of vagina smears presents results to unacceptable low sensitivity • Requires a trained personnel	[7,14,24,25]
02	Biopsy	• Much more specific • Gold standard in confirming FGS cases	• Involves a painful sample collection • Some eggs might be missed out when collecting biopsy samples • Require a trained personnel • Biopsy increases the risk of STI acquisition if intercourse is conducted before healing • Not field deployable	[19,26]
03	Colposcopy	• There is freely available pocket atlas from the WHO that provide clear guidelines on how to interpret colposcopy findings • Handheld colposcopy can be operated by primary healthcare staffs	• Traditional colposcopy is expensive, and requires a trained personnel • Low specificity • Not field deployable • Manipulation and rotation of speculum to visualize all the fornices and vagina walls might be painful • It can only be done in women who are sexually active and not virgin girls	[4,8,26–30]
04	Circulating anodic antigen (CAA)-based assays	• Can be used to screen urogenital schistosomiasis	• Demonstrating *Schistosoma* antigens does not necessarily indicate FGS	[7,24,27,31]
05	PCR and qPCR	• Allows self-collection of genital samples	• Not field deployable • Genital samples offer lower sensitivity in detecting *Schistosoma* DNA compared to urine samples •Require trained personnel	[7,26,27,31–34]
06	Isothermal amplification assays	• Allows self-collection of genital samples • Can be field deployable	• There is no enough data to support their performance • Genital samples can lower sensitivity in detecting *Schistosoma* DNA compared to urine samples	[7,26,35–37]

Women with FGS may exhibit symptoms and receive diagnoses through the visual inspection of characteristic lesions on the cervix and vagina, despite not excreting schistosome eggs in their urine [2,4]. Usually, FGS diagnosis relies on demonstrating FGS lesions using biopsy and colposcopy approaches. Genital biopsy assay is much more specific, but needs trained personnel, and sample collection procedures increases the likelihood for sexual transmitted diseases, especially if a female undergoes intercourse before healing. Colposcopy can also be used to diagnose FGS in vagina, cervix, and vulva, but most healthcare professionals are unable to identify FGS lesions [4,38]. However, the WHO in 2015 designed a pocket atlas to aid health professionals to diagnose the disease by visualizing egg-induced lesions patches and abnormal blood [4]. Sandy patches in female genital indicate chronic FGS, while rubbery papules potentially indicate acute FGS infection [8]. The applicability of this method faces some challenges: one, the method can only be used to diagnose FGS in sexually active and non-virgin females, and the stigmatization makes it difficult for some women to undergo pelvic examination. Two, colposcopy requires trained personnel, and in some cases, false results occur when FGS is affecting the genital parts like uterus, ovary, and fallopian tube, which cannot undergo examination by this approach.

In order to overcome challenges with biopsy and colposcopy approach, some studies have evaluated the potential of PCR-based assays and isothermal amplification of *Schistosoma* DNA [32,33,35,39]. Most of these methods have been repeatedly reported in research setting, most of them being far from being deployed to the field settings. Isothermal amplification assays could potentially be field deployable due to the possibility to amplify *Schistosoma* DNA in field settings using a single temperature, but optimization and validation is required to ensure that the assays can correctly detect *S. haematobium* DNA in self-collected swabs [34,35].

## Knowledge gap about FGS

There have been reports of FGS co-occurrence with other diseases like HIV/AIDS, cervical cancer, syphilis, and gonorrhea [9,11,26]. Particularly in Africa, FGS does not receive the attention it deserves [40–42]. The disease is not included in medical books or health curriculums, leading to a knowledge gap among communities and healthcare professionals [11,40,43]. In most cases, there is no enough expertise and facilities available for diagnosing the disease [13,44]. Healthcare practitioners often confuse the symptoms of FGS with other sexually transmitted diseases, only suspecting, diagnosing, and treating the disease when patients do not respond to other therapies [45]. Consequently, the underdiagnosis mistreatment of FGS in young ladies and females, particularly in rural areas of sub-Saharan Africa, leads to a silent pandemic of drug resistance, the development of preventable infertilities and cervical cancers, and an increased risk of HIV due to the stigma associated with FGS testing [9,19]. Several studies in Tanzania, the country with the second highest prevalence of schistosomiasis, have also revealed that the majority of healthcare practitioners and patients are not aware that urogenital schistosomiasis can impact female reproductive systems [21,44]. Another recent study conducted in Kimpese region, in the Democratic Republic of Congo (DRC) revealed that 91% of respondents knew about schistosomiasis, and 45% among them were unaware about FGS [46]. The knowledge gap, particularly among healthcare practitioners and family partners, contributes to the ongoing stigmatization that facilitates mistreatment or makes patients seek treatment from traditional healers [21]. In some cases, for instance, married women have to go through painful sexual intercourse to fulfill the sexual desire of their husbands so that they can avoid being abandoned, extra-marital affairs, or being told they are prostitutes [20].

## Treatment

The treatment and control of FGS rely on current guidelines for controlling and eliminating schistosomiasis without rigorous clinical evidence of the presence of FGS [24]. These guidelines promote the use of praziquantel, which is effective against adult schistosomes, but not juvenile schistosomes and eggs. Furthermore, the current WHO guidelines, which advocate for yearly preventive chemotherapy with praziquantel for all individuals over 2 years old, especially those with a *Schistosoma* prevalence of at least 10% and primary school-aged children, present shortcomings in FGS eradication strategies. First, neglecting FGS in less prevalent areas facilitates the development of chronic FGS, putting those affected at risk of acquiring preventable cervical cancer and infertility. Equally important, focusing on the younger group leaves most adult women untreated, leading to a continued FGS transmission among communities [19,30,47]. Herein, the most practical ways to combat FGS would be to integrate the disease into routine sexual and reproductive health services so that young ladies and women who are affected by the disease receive early treatment [40,48].

## *S. haematobium* hybridization: A potential and emerging threat to FGS molecular diagnosis.

**Fig 4:**
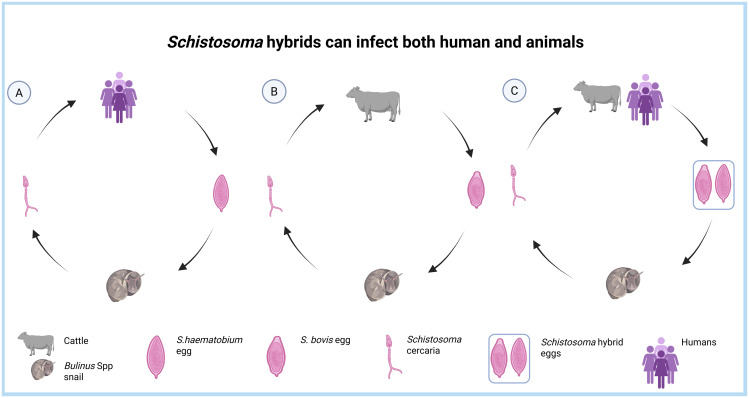
Infection cycles and hybridization of *Schistosoma* species. **A**: traditional infection cycle of *S. haematobium* in humans; **B**: traditional infection cycle of *S. bovis* in cattle, and **C**: zoonotic schistosomiasis infection cycle of *S. haematobium* and *S. bovis* hybrids in humans and cattle. Created in BioRender.Com.

Hybridization is a novel interspecies genetic material acquisition process that occurs when two related species interbreed (Fig 4) [49]. *Schistosoma* hybridization is an emerging public health concern and threat to schistosomiasis elimination strategies [16,50,51]. Several countries, mostly in Africa, have reported genetic hybridization between *S. haematobium* and livestock schistosomes, particularly *S. bovis* and *S. curassoni* [15]. Researchers have reported *S. haematobium* × *S. bovis* hybrids, the most studied hybrid, in Cameroon, Nigeria, Niger, Mali, Senegal, Ivory Coast, and Benin [15,16,49,52–54].

These hybrids have been isolated from domestic animals, definitive human hosts, and the intermediate *Bulinus* Spp snails [17]. A study in Cameroon reported that 11.3% of *S. haematobium* × *S. bovis* hybrids from miracidia collected from schoolchildren’s urine were found in ten sites in the country [50]. Based on the Cox1 profile, Onyekwere *and colleagues* (2022) found that Nigeria, the most prevalent country for schistosomiasis, has a country-wide minimum proportion of 89% prevalence of *S. bovis* × *S. haematobium* hybrids and almost equal proportion among the study sites [15]. Another study in Ivory Coast reported that 97.8% of miracidia isolated from children from 11 sites were *Schistosoma* hybrids [53].

The hybridization pattern remains unclear, but a few studies suggest that the pattern in *S. haematobium* × *S. bovis* may be due to gene transfer from the livestock schistosome, *S. bovis*, to *S. haematobium* [15,55]. These studies argue that *S. haematobium* hybridization occurs in pastoralist areas where there is fresh water, and animals and humans coexist [56,57]. One study reported that *S. haematobium* isolates harbor signals of introgression from *S. bovis*, and these isolates differed from each other due to conserved introgressed genomic regions originating from *S. bovis*, which could be the result of either local adaptive processes or genomic drift. Another study in Ivory Coast isolated pure *S. haematobium*, *S. haematobium* hybrids, and pure *S. bovis* in children, indicating that *S. bovis*, a bovine schistosome, is capable of infecting humans [58]. However, most of these studies have investigated hybridization based on the Cox 1 and ITS gene profiles, which may have resulted in underestimating the actual prevalence [15,52,53,58]. Future studies should therefore focus on whole genome sequencing of schistosomes from different geographical areas to map the real transmission patterns and develop much more markers that indicate hybrid schistosomes [56,59].

The current FGS diagnostic approaches based on the visual examination of *Schistosoma* eggs and vaginal lesions are much more specific but not field deployable; they do not allow self-sample collection, involve painful procedures, and are therefore not widely accepted. As a result, researchers are focusing on developing and validating isothermal-based assays for *S. haematobium* DNA amplification using vaginal samples to overcome challenges caused by the use of biopsy and colposcopy approaches. However, recent reports on the occurrence of *S. haematobium* hybrids further complicate these initiatives [15,55]. *Schistosoma* hybrids not only can infect humans and livestock, but also alter the genetic diversity and population structure of schistosomes [15,50,59], which can significantly affect the genetic diversity, egg morphology [14], and subsequently, FGS diagnosis. For example, a recent study by Juhász *and colleagues* (2024) reported to have isolated pure *S. haematobium* from cattle, and the methodology was based on *Cox1* and *ITS* gene profile [57]. Herein, isolating a human schistosome in cattle indicates that it is either animals could potentially reserve and transmit human schistosomiasis or the urgent need for much more markers in deciding whether the schistosomes are pure or hybrids. Several studies as well, have relied on PCR-based assays for diagnosing FGS by targeting ITS gene profile [31,32]. Assuming that some samples had *Schistosoma* hybrids with their ITS gene profile mutated, it is most likely that some *Schistosoma*-positive samples were designated negative.

## Conclusion

The current approaches to diagnosing urogenital schistosomiasis may not adequately identify *S. haematobium* hybrids. Microscopy, the gold standard for identifying *Schistosoma* species, cannot detect mutations in *Schistosoma* hybrids. Most studies have estimated the prevalence of *Schistosoma* hybrids based on the Cox and ITS profiles, which again might have underestimated the actual prevalence of *S. haematobium* hybrids in humans and animals, especially if genetic hybridization in some species affects conserved regions other than the most commonly used. Herein, the underestimation of the actual *Schistosoma* prevalence can significantly affect the current schistosomiasis elimination strategies, which advocate for yearly preventive chemotherapy with praziquantel for all individuals over 2 years old, especially those with a *Schistosoma* prevalence of at least 10%.

Still, molecular assays continue to be the most practical method for detecting *S. haematobium* hybrids. In line with the 2022 WHO call on the development and evaluation of existing molecular diagnostic assays for schistosomiasis, we recommend that new assays for *S. haematobium* in general urogenital schistosomiasis or FGS should incorporate more than a single target or should target identical multi-repeat regions, which are much more resilient to a few mutations.

## Key learning points

1. The diagnosis of FGS remains challenging as there is no widely accepted reference assay.
2. The treatment and control of FGS rely on current guidelines for controlling and eliminating schistosomiasis without rigorous focus on clinical evidence of the presence of FGS.
3. To eliminate FGS-related complications, there is a need to expand schistosomiasis elimination strategies to provide preventive chemotherapy to young ladies and women of all age groups, including those who are not in school.
4. Most healthcare professionals and communities have a knowledge gap about FGS, leading to stigmatization, underdiagnosis, mistreatment, and the continued incidence of preventable infertilities and cervical cancers among young ladies and women in areas where *S. haematobium* is prevalent.
5. Several African countries have reported hybridization between *S. haematobium* and other livestock schistosomes, which could significantly impact FGS elimination strategies.

## Selected publications

**Table:** 

No	Publication
01	Rossi B, Previtali L, Salvi M, Gerami R, Tomasoni LR, Quiros-Roldan E. Female Genital Schistosomiasis: A Neglected among the Neglected Tropical Diseases. Microorganisms. 2024;12(3).
02	Onyekwere AM, Rey O, Allienne JF, Nwanchor MC, Alo M, Uwa C, *Et al*. Population Genetic Structure and Hybridization of *Schistosoma* haematobium in Nigeria. Pathogens. 2022;11(4):1–15.
03	WHO. WHO GUIDELINE on control and elimination of human schistosomiasis. 2022. 142 p. Available from https://www.who.int/publications/i/item/9789241509299
04	Mazigo HD, Samson A, Lambert VJ, Kosia AL, Ngoma DD, Murphy R, *Et al*. “We know about schistosomiasis but we know nothing about FGS”: A qualitative assessment of knowledge gaps about female genital schistosomiasis among communities living in *Schistosoma* haematobium endemic districts of Zanzibar and Northwestern Tanzania. PLoS Negl Trop Dis [Internet]. 2021;15(9):1–25. Available from: http://dx.doi.org/10.1371/journal.pntd.0009789
05	Orish VN, Morhe EKS, Azanu W, Alhassan RK, Gyapong M. The parasitology of female genital schistosomiasis. Curr Res Parasitol Vector-Borne Dis [Internet]. 2022;2(May):100093. Available from: https://doi.org/10.1016/j.crpvbd.2022.100093
